# The Contribution of Three-Dimensional Power Doppler Imaging in the Preoperative Assessment of Breast Tumors: A Preliminary Report

**DOI:** 10.1155/2009/530579

**Published:** 2009-08-24

**Authors:** K. Kalmantis, C. Dimitrakakis, Ch. Koumpis, A. Tsigginou, N. Papantoniou, S. Mesogitis, A. Antsaklis

**Affiliations:** 1st Department of Obstetrics and Gynecology, Alexandra Hospital, Athens University Medical School, Athens, Greece

## Abstract

*Purpose*. The aim of this study was to determine the value of 3D and 3D Power Doppler sonography in the detection of tumor malignancy in breast lesions and to find new diagnostic criteria for differential diagnosis. *Methods*. One hundred and twenty five women with clinically or mammographically suspicious findings were referred for 3D Power Doppler ultrasound prior to surgery. Histological diagnosis was conducted after surgery and compared with ultrasound findings. Sonographic criteria used for breast cancer diagnosis were based on a system that included morphological characteristics and criteria of the vascular pattern of a breast mass by Power Doppler imaging. *Results*. Seventy-two lesions were histopathologically diagnosed as benign and 53 tumors as malignant. Three-dimensional ultrasound identified 49 out of 53 histologically confirmed breast cancers resulting in a sensitivity of 92.4% and a specificity of 86.1% in diagnosing breast malignancy (PPV: 0.83, NPV:0.94). *Conclusions*. 3D ultrasonography is a valuable tool in identifying preoperatively the possibility of a tumor to be malignant.

## 1. Introduction

Mammography is the only well established screening method for the detection of breast cancer. Today, the aim of differentiating malignant from benign breast tumors and diagnosing early breast cancers is more accurately fulfilled with the addition of ultrasound scan. It has been reported that combined mammography and sonomammography to women with dense breasts or other high-risk factors, result to a higher sensitivity in breast cancer diagnosis [1, 2]. Modern, high resolution breast ultrasonography presents the ability of depicting even mammographically occult, asymptomatic breast cancers [3]. Ultrasonographic sensitivity is not confined of dense breast tissue and it is an extremely useful modality for breast imaging in young and pregnant women [4, 5].

Real-time ultrasonography experience over the years and high quality imaging technology made possible the establishment of certain criteria of tumor examination. The assessment of the tumor shape, orientation, the definition of tumor margins and the presence or absence of retraction pattern remain the most acceptable and sensitive differentiating markers of malignancy [6]. 

3D mammasonography is the most recent development in breast ultrasound imaging, providing additional aspects to conventional 2D sonography. New superior diagnostic information such as the ability to study a breast mass and the surrounding tissue in three orthogonal planes with high frequency transducers, overcomes anatomical limitations which restrict the number and orientation of the scanning planes and allows an objective analysis of breast tumor morphology and angiogenesis [7]. 

New Doppler methods, such as Power Doppler, obtain better results that conventional Doppler in detecting vascularity of solid breast masses and allow more detailed depictions of vessel structure and tissue vascularity in slow and poor flow areas. Neovascularization of a carcinoma with an irregular vascular pattern, arteriovenous shunts and missing vessel autoregulation—in contrast to normal breast tissue vessels—aims to differentiate between benign and malignant breast tumors [8]. However, the proof of extent of tumor vascularity is not a sufficient criterion for differential diagnosis and identification of fast growing tumoral capillaries has not gained clinical relevance until now [9, 10].

3D Ultrasound facilities and 3D Power Doppler are additional and no replacing modalities to breast ultrasound imaging. Today, there is no certain data available of superiority of 3D techniques over the classic sonomammography in breast cancer diagnosis [11, 12]. Nevertheless, with these “high-end” techniques, the final imaging is improved, adding this way a diagnostic advantage to clinical or mammographical suspicion or exclusion of malignancy and proper treatment plan. However, diagnosis may remain uncertain for some breast tumors that exhibit variable sonographic characteristics due to differences in histological type, histological grading and tissue components within the tumors.

In order to avoid unnecessary biopsies but without missing cancers, or to better plan an operation, new diagnostic procedures are sought. The combined use of mammography and 3D imaging may have clinical utility in the early detection of breast cancer. The goal of this study was to estimate the diagnostic value of this new method in distinguishing benign from malignant breast lesions.

## 2. Materials and Methods

One hundred and fifty three patients with palpable breast masses or abnormal mammograms underwent ultrasound and 3D Power Doppler ultrasound scan, in order to differentiate malignant from benign lesions preoperatively. Patients were recruited consequently from April 2007 to November 2007 and were referred to our breast cancer center for further evaluation of the mass and treatment. Twenty eight patients were not eligible and were excluded from the study because clinically or mammographic lesions were not visible on ultrasound or, they had cysts or, tumors were too big for 3D ultrasound or, patients gave no informed consent. The remaining 125 women, aged 18 to 70 years (mean age 45 years), had solid tumors and after clinical evaluation and ultrasonography, they were treated surgically with open excisional biopsy. In this study we compare primary ultrasonographic characteristics and diagnoses before surgery to histopathological findings after surgery. No data of preoperative cytologic results is included.

For the ultrasound scans we used a 3D ultrasound volume transducer 5–13 Mhz with a 30° volume sector, Voluson 730 Pro, General Electric. Ultrasound examination was performed with patients in supine position with elevated arms by two sonographers experienced in breast ultrasound. Ultrasound examiners were not aware of clinical and mammographic findings. Once the region of interest had been identified the volume box was superimposed and the ultrasound probe was kept steady. The volume mode was switched on and the 3D ultrasound volume was generated by the automatic rotation of the mechanical transducer through 360°. The acquisition time ranged between 2–4 seconds depending on the size of the volume box. Three perpendicular planes were displayed simultaneously thus enabling better understanding of the morphology of breast lesion. Evaluation of the stored volumes took 10–15 minutes, depending on the number of slices, rotation angle and rendering modes used. Since the number and orientation of reformatted planed are not limited meticulous evaluations of numerous sections thorough the tumour becomes possible.

Ultrasound examination evaluated suspicious lesions for their shape, orientation, margins, the echogenicity of the lesion and the surrounding tissue, echotexture, acoustic transmission, pattern, and vessel's architecture (Table 1). Additionally, Pourcelot resistance index (RI) was used as the calculated measurements of the flow velocity wave form (systole minus diastole divided by systole). The Doppler variable used for diagnosing breast malignancy was RI ≤ 0.42 obtained from the periphery or from central areas of the tumor.

Benign ultrasound characteristics as round-oval shape and a long axis parallel to skin were scored by 0 and malignant characteristics as inhomogenous echotexture and a star pattern in the coronary plane were scored by 2. The scoring system was used for each one of the above criteria as it is shown in Table 1. After completing ultrasound examination, scores for each parameter were added to make a sum for each patient. The bigger the number the higher is the possibility for malignancy. We used 6 as the cutoff point of malignancy. Final score by combined ultrasound imaging less than 6 (<6) favors a benign lesion while a score of 6 or more (≥6) assessed as predicting malignancy.

The 3D sonographic characteristics used to differentiate benign from malignant lesions were based on a specific scoring system including morphologic characteristics and criteria of the vascular pattern of a breast mass [13–19].

The final ultrasound estimation scores were compared with histopathology reports of the tumor specimens.

Distributional properties at 3D and 3D Power Doppler outcomes with respect to the ten criteria that were investigated by chi-square statistics (*x* ^2^-test).

All patients included in this study were properly informed before signing a written consent and the study were approved by the ethical committee of the hospital.

## 3. Results

**Figure 1:**
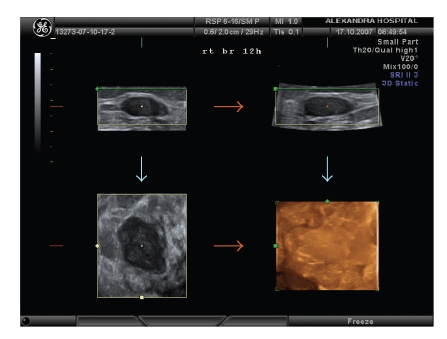
3D pictures of a fibroadenoma.

**Figure 2:**
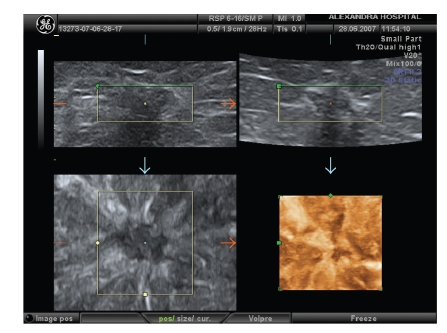
3D reconstruction of an invasive ductal carcinoma angulated with inhomogenous echotexture and retraction pattern.

Seventy-two patients were diagnosed to have benign tumors (adenoma, fibroadenoma, papilloma, phyllodes tumor, radial scar, and lipoma) and 53 malignant (mainly ductal and lobular, few papillary, mucinous, medullary and tubular carcinomas), according to histopathology reports after surgery. The most common benign breast tumor was fibroadenoma (Figure 1) while the most common malignant tumor was ductal carcinoma (Figure 2). The combined use of 3D ultrasound and 3D Power Doppler identified 66 breast tumors as benign (score <6) and 59 tumors as malignant (score ≥6) (Table 2). The 3D and 3D Power Doppler ultrasound gave 10 false positive results (2 adenomas, 2 fibroadenomas, 3 radial scar, and 3 papillomas) and misdiagnosed 4 carcinomas (1 ductal, 1 papillary, 1 medullary, and 1 tubular carcinomas). Cutoff score greater or equal to 6 was associated with a high potential for breast malignancy.

**Figure 3:**
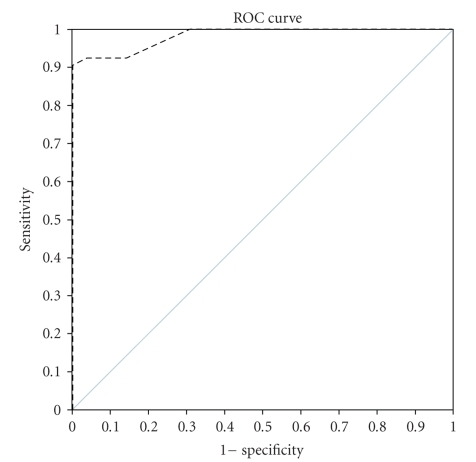
High diagnostic capability of 3D ultrasound in differentiating benign from malignant breast tumors.

In our series, the accuracy of 3D and 3D Power Doppler ultrasound in assessing a suspicious mammography lesion appeared high with a sensitivity of 92.5% and a specificity of 86.1%. The positive and negative predictive values were 83.1% and 93.9%, respectively. Performance classification was assessed by Receiver Operating Characteristic curve (ROC): a graphical plot of the sensitivity versus the false positive rate, (Figure 3).

**Figure 4:**
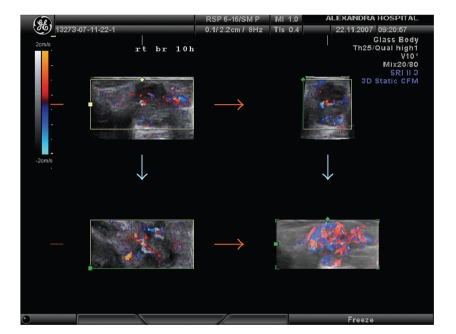
3D Power Doppler imaging of a malignant tumor with chaotic vessels' architecture and complex branching pattern.

Combined 2D and 3D Power Doppler sonographic features of all patients are summarized in Table 3. Distributional properties at 3D and 3D Power Doppler outcome with respect to the ten criteria were investigated by chi-square statistics (*x* ^2^-test). Distinct differences in imaging between malignant and benign tumors were found statistically significantly in all of the ten criteria used: benign tumors appeared in a round/oval shape in 52 cases (72.2%), with the horizontal diameter wider than the vertical in 49 cases (68.1%), with smooth defined margin in 70 cases (97.2%), with no surrounding echogenicity to all 72 cases (100%), with hyperechoic echotextures in 63 cases (87.5%), and with homogenous echotextures in 37 cases (51.4%). Acoustic transmission was present in 36 cases (50%) and compression pattern was observed in 69 cases (95.8%). Malignant tumors, on the contrary, appeared in 3D with chaotic vessel architecture with complex branching pattern (Figure 4) in 28 cases (52.8%). The R.I. value was lower than 0.42 in 2 cases of benign (2.8%) and in 24 cases of malignant tumors (45.3%).

## 4. Discussion

Mammographic screening is the only screening method proven to decrease breast cancer mortality. It is understood that early detection of small subclinical lesions and subsequent surgical treatment is the cornerstone of successful screening. Throughout the years, additional imaging modalities to mammography have been inserted to clinical practice. One of these is the ultrasound scan of the breasts which in the beginning helped to differentiate benign cysts from solid masses. Today, development of ultrasound technology and high quality of modern 2D breast sonography resulted in imaging detailed anatomical structures, and nonpalpable pathological lesions [20]. Mammasonography is considered a worthwhile adjunct to mammography in differentiating benign from malignant lesions [21, 22]. In a recent study, addition of ultrasound to mammographic screening resulted in a 55% increase in breast cancer diagnosis in a high risk population [23]. These results are expected to reflect in a further reduce of mortality rates in future trials.

Ultrasound screening shows the potential to depict early, node negative, small sized breast cancers that sometimes escape mammography illustration and its performance seem accurate in dense parenchyma [24–26]. However, Moon et al. reported that sonography can depict small or non-palpable lesions but its accuracy in diagnosis of malignancy is low [27].

With increasing experience in breast ultrasound imaging, criteria of differentiation established to increase the accuracy and achieve a consensus among radiologists when describing breast abnormalities [13, 28]. Sonomorphological parameters of breast tumors such as shape, tumor margins, internal echogenicity, echotexture distribution and posterior echo have been shown to be valuable in the differential diagnosis between benign and malignant breast tumors [6, 29].

The shape of a solid tumor is one of the primary features studied for differential diagnosis. Oval or royal shape is considered as a benign characteristic of a solid mass [30]. In our patients a round oval shape was found in 72.2% of benign breast tumors and only in 1.9% of malignant tumors, while irregular shape was found in 79.2% of malignant tumors. In former studies the oval/round shape characterizes benign tumors in 82,5 to 86% and is found in 24 to 42% of malignant tumors, depended on the study [30]. This increased accuracy in our study may be partly explained by the high quality imaging pictures we have today in breast ultrasonography.

Appearance of margins and tumor orientation represent two of the most important tumor features [31]. Spiculated or not defined margins in combination with abnormal shape and nonparallel orientation of the tumor are highly susceptive of malignancy. Speculated margins, in fact, are suggesting of an infiltrating process and nonparallel orientation may be the result of abnormal growth of a tumor through tissue planes as cancer develops. In contrast, circumscribed, defined margins and parallel orientation (with the wide axis larger or equal to the tall axis) of the lesion are predictive of a benign diagnosis and of a tumor which growths without interfering to the surrounding tissues [29]. In our study, 75% of breast cancers and 2.8% of benign tumors had ill defined margins. The nonparallel orientation was found in 83% of cancer cases and in 2.8% of benign tumors, giving a sensitivity of 0.83 and a specificity of 0.97. These increased sensitivity rates may be the result of the high quality images of real time ultrasonography in combination with an increased clinical or mammographical suspicion of the examined lesion that entailed to increased examiner's attention to the tumor characterization.

Invasive tumors tent to appear sonographically inhomogenous. Margins are echo-rich because of the variety of tissue components expressed by the tumor and the surrounding infiltrated parenchyma. Homogenous echo-poor center is the result of fibrohyalinosis. Dorsal shadowing frequently depict as a consequence of ultrasound energy absorption through tumor [32, 33]. Tumor echogenicity, echotexture and presence of posterior shadowing are considered important sonographic features in evaluation of breast lesions [31]. The vast majority of benign tumors in our sample are hyperechoic and most of malignancies have inhomogenous echotexture. These criteria are reported to be important sonographic features suggesting a malignant tumor and present in 72–97% of breast carcinomas. Also, in our patients, absence of acoustic transmission was present in almost 85% of malignant tumors. In contrast, it is reported in the literature that an internal echo pattern and echogenicity have a low discriminating value.

3D ultrasound is one of the most recent developments in breast imaging, providing additional aspects to conventional 2D sonography such as the ability to study a breast mass and the surrounding tissues in three orthogonal planes. Exceeding the limitation of scanning only 2D planes, 3D ultrasound imaging modality access the imaging of coronal, transversal and sagittal planes simultaneously. Transverse and sagittal plane of the typical 2D ultrasound allow evaluation of architecture distortions like connective tissue disruption and changes of shape and orientation. For stellate tumors or carcinomas with diffuse pattern of growth, the malignant infiltration is impressively apparent in coronal plane by 3D, as a retraction pattern. On the contrary, benign lesions are associated with a compressive pattern. These two patterns show high specificity and sensitivity (0.938 and 0.914, resp.) and high predictive values (positive 0.869, negative 0.960) when used as criteria for differential diagnosis [7, 34]. Even in small stellate carcinomas with diameter smaller than 1 cm, the retraction pattern is visible in the coronal plane. In the coronal plane, architectural distortion may be the only imaging finding of a lobular carcinoma that is not apparent as a mass in mammography or conventional 2D sonography [7, 12, 35]. In our series, retraction/stellate pattern was apparent in 88,7% of malignant cases (sensitivity 0.89, specificity 0.96, positive predictive value 0.96, negative predictive value 0.92) which is close to that reported in the literature.

3D ultrasonography offers a clear imaging of lesions with abnormal shapes, like complex fibroadenomas with lobulation on their surfaces or irregular aspects. Additional information for the lesion can be collected, like the spatial distribution of echo texture, related to an echodifferent area within a fibroadenoma such as calcification. Another advantage of this method is that 3D multiplanar image is independent of the diameter of the long or short axis of the lesion and angulations, resulting in objective measurement of the tumor. With 3D reconstruction and display modes as the niche mode, transparent maximum and surface mode, extra data can be collected. These modalities allow the visualization of the entire nipple area and the retromamillary region in one volume obtaining full diagnostic information. It also demonstrates reliably the ductal anatomy and the pathology of intraductal components. Sonographic evaluation of the retromammilary region is considered as the most accurate imaging modality [36]. 

Vascularisation of a breast lesion is possible to be examined using a 3D Power Doppler technique. Neovascularization, irregular vascular pattern, arteriovenous shunts and missing vessel autoregulation are converging to carcinoma diagnosis. Benign lesions and normal breast tissue lack of these characteristics [10]. Although the morphological pattern of tumor vessels can be assessed by a 2D Doppler sonography, 3D Power Doppler provides the advantage of visualization of the entire lesion. Additionally, 3D reconstructions of color volume data offers the ability to examine the vessel distribution and potential irregularities of vessel shape. The 3D display of power flow imaging data can be rotated to facilitate the study of the architecture of tumor vessels in various projections. Views of the value in different projection of malignant lesions clearly reveal the feeding vessel and the entire neovascular network intra- and peritumoraly [10]. Irregular vessel calibers, penetration of the tumor margins and irregular vascularisation are suspicious for malignancy [9].

3D Power Doppler is considered as a very sensitive method for detecting blood flows of low-velocity and low-volume. 3D Power Doppler technique advantages include the detection of even minimal blood flow, the reliable analysis of vessel architecture, the vascularisation pattern extend and the definition of the number of vascular poles.

The sensitivity of 3D Power Doppler method is reduced in some cancers that have undetectable blood flow values and in some proliferative benign lesions that may present with increased blood flow and vascularisation. In our study, 3D Power Doppler depicted abnormal vascularisation in malignant tumors with a relatively low sensitivity (0.53), but with a high specificity of 0.97.

Continuous wave and duplex ultrasound studies have shown that in carcinomas the blood flow is more intense than in benign lesions. Resistance Index [R.I = (systolic − diastolic)/systolic] study shows no particular difference between malignant and benign tumors [7, 37]. There is inconsistent information about resistance index and pulsatility index in breast cancer. This could be explained by the chaotic, irregular vascular pattern and the presence of abnormal vessels in malignant tumors with decreased intratumoral blood flow resistance. On the other hand, loss of intratumoral tissue elasticity may lead to an increase in blood flow resistance. The probability to detect a low min R.I. is strongly influenced by the number of vessel of cross sections. So it is not surprising flat most published studies about differentiation between benign and malignant tumors have found significantly lower min R.I. in the average better per fused malignant tumors [38]. Based on Uurjan's color Doppler scale we considered and adopted cutof value of R.I.(I < 0,42) for a malignant breast tumor [39]. In our study, R.I. was low (<0.42) in 45.3% of malignant cases (sensitivity 0.44).

In the present study, the combined use of 3D and 3D Power Doppler imaging identified a breast mass as malignant tumor when it appeared with the following characteristics: irregular shape, abnormal orientation (taller than wider), ill defined margin, inhomogenous echotexture, absence of acoustic transmission, retraction or star pattern, chaotic vessels architecture and sometimes with an RI < 0.42.

Finally, the combined scoring system for sonographic criteria, that we used for this study have correctly excluded malignancy in 91.2% of the cases. False positive rate though was high and, 11.3% of the cases had an ultrasound cancer diagnosis that was not confirmed histopathologically (Table 3). As it is reported, preoperative or screening sonomammography shows an increased sensitivity in breast cancer diagnosis. Modern modalities improve the final tumor imaging and perhaps the proper treatment choice but, they still involve a substantial false positive rate that can result to unnecessary breast biopsies [2, 3, 40].

## 5. Conclusion

3D Ultrasound is an effective tool in evaluating the morphology of breast masses and plays a significant and supplementary role to mammography, especially in dense mammary tissue. This new technique, based on a specific scoring system, gives reliable information and characterizes the breast tumors. It gives more comprehensive information on anatomical details and pathological structures and offers a new diagnostic aspect for the differentiation between benign and malignant breast lesions. The Power Doppler findings should be regarded as additional diagnostic features to the sonographic evaluation of breast lesions, besides the already established criteria, with the potential to improve differential diagnosis. However, in view of the small number of patients with breast tumors in our study, these observations should be considered as preliminary. Further studies are needed to establish the role of 3D ultrasound in tumor detection and differential diagnosis and to determine treatment options.

## Figures and Tables

**Table 1:** 3D Ultrasonographic criteria for breast tumors.

(1) Shape	Round/oval	0
	Lobular	1
	Irregular	2

(2) Orientation	Wider than tall (W > T)	0
	Not clear (W = T)	1
	Taller than wide (W < T)	2

(3) Margin	Well defined	0
	ILL defined (microlobulated)	2

(4) Surrounding echogenicity	Hypoechogenic regular (pseydocapsule)	0
	Echogenic irregular	2

(5) Echogenicity	Hyperechoic	0
	Hypoechoic	2

(6) Echotexture	Homogenous	0
	Inhomogenous	2

(7) Acoustic transmission	Present (enhancement)	0
	Absent	2

(8) Pattern	Compression pattern	0
	Retraction/star pattern	2

(9) Vessels architecture	Linear	0
	Chaotic-complex	1

(10) R.I.	≥0.42	0
	<0.42	1

**Table 2:** 3D Ultrasound findings compared with histopathology reports.

	3D Ultrasound		Histopathology
	Diagnosis	Histologicaly confirmed preoperative diagnoses	
Benign	66	62 (86,1%)	72
Malignant	59	49 (92,4%)	53
Total	125	111 (88,8%)	125

**Table 3:** 3D outcome according to the sonographic criteria used in our breast tumor patients.

			Benign		CA	
		*N*	%	*N*	%	*P*
Shape	Round/oval	52	72.2%	1	1.9%	<.0001
	Lobular	20	27.8%	10	18.9%	
	Irregular	0	0%	42	79.2%	
Orientation	(W > T)	49	68.1%	4	7.5%	<.0001
	(W = T)	21	29.2%	5	9.4%	
	(W < T)	2	2.8%	44	83.0%	
Margin	Well defined	70	97.2%	13	25%	<.0001
	ILL defined	2	2.8%	40	75%	
Surrounding echogenicity	Hypoechogenic regular	72	100.0%	24	45.3%	.002
	Echogenic irregular	0	0%	29	54.7%	
Echogenicity	Hyperechoic	63	87.5%	33	62.3%	.002
	Hypoechoic	9	12.5%	20	37.7%	
Echotexture	Homogenous	37	51.4%	12	22.6%	<.0001
	Inhomogenous	35	48.6%	41	77.4%	
Acoustic transmission	Present	36	50.0%	8	15.1%	<.0001
	Absent	36	50.0%	45	84.9%	
Pattern	Compression pattern	69	95.8%	6	11.3%	<.0001
	Retraction/star pattern	3	4.2%	47	88.7%	
Vessels architecture	Linear	70	97.2%	25	47.2%	<.0001
	Chaotic-complex	2	2.8%	28	52.8%	
R.I.	≥0.42	70	97.2%	29	54.7%	<.0001
	<0.42	2	2.8%	24	45.3%	
